# Glycomics Approaches for the Bioassay and Structural Analysis of Heparin/Heparan Sulphates

**DOI:** 10.3390/metabo2041060

**Published:** 2012-11-28

**Authors:** Tania M. Puvirajesinghe, Jeremy E. Turnbull

**Affiliations:** 1 Centre de Recherche en Cancérologie de Marseille, Inserm U1068, CNRS UMR7258, Institut Paoli-Calmettes, 10039 Marseille, France; 2 Centre for Glycobiology, Department of Biochemistry and Cell Biology, Institute of Integrative Biology, The University of Liverpool, Liverpool, L69 7ZB, UK; Email: j.turnbull@liverpool.ac.uk (J.E.T.)

**Keywords:** heparan sulphate, heparin, glycomics, glycan arrays, glycoarrays, glycobioarrays

## Abstract

The glycosaminoglycan heparan sulphate (HS) has a heterogeneous structure; evidence shows that specific structures may be responsible for specific functions in biological processes such as blood coagulation and regulation of growth factor signalling. This review summarises the different experimental tools and methods developed to provide more rapid methods for studying the structure and functions of HS. Rapid and sensitive methods for the facile purification of HS, from tissue and cell sources are reviewed. Data sets for the structural analysis are often complex and include multiple sample sets, therefore different software and tools have been developed for the analysis of different HS data sets. These can be readily applied to chromatographic data sets for the simplification of data (e.g., charge separation using strong anion exchange chromatography and from size separation using gel filtration techniques. Finally, following the sequencing of the human genome, research has rapidly advanced with the introduction of high throughput technologies to carry out simultaneous analyses of many samples. Microarrays to study macromolecular interactions (including glycan arrays) have paved the way for bioassay technologies which utilize cell arrays to study the effects of multiple macromolecules on cells. Glycan bioassay technologies are described in which immobilisation techniques for saccharides are exploited to develop a platform to probe cell responses such as signalling pathway activation. This review aims at reviewing available techniques and tools for the purification, analysis and bioassay of HS saccharides in biological systems using “glycomics” approaches.

## 1. Introduction—The Complexity and Challenges Associated with Glycomics

**Figure 1:**
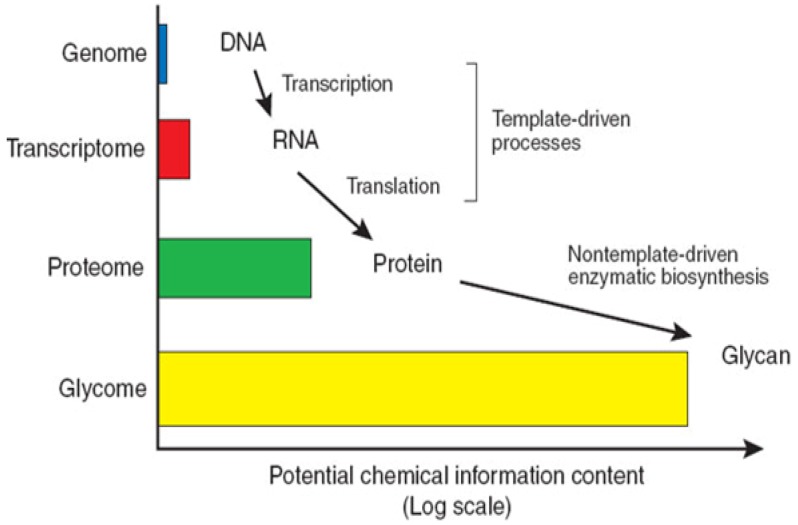
The complexity of the glycome. The glycome is defined as the complete set of glycan structures expressed at a particular time and spatial position in specific cells, tissues or organisms. Therefore the complexity of structures and the potential chemical information of the glycome far exceed that of the genome and proteome. Figure taken from [1].

Following the success of naming certain subdivisions of sciences with the suffix “omics”, the term “glycomics” was coined to describe the comprehensive study of the glycome, with the glycome itself characterised as the complete set of glycan structures expressed at a particular time and spatial position in specific cells, tissues or organisms [1]. This review provides a summary of the techniques used for the structural analysis, bioinformatic interpretation of data and methods used for the bioassay of HS and other GAGs glycans. The Human Genome Project has concluded that only 30,000–50,000 genes account for human complexity. This emphasizes the exquisite complexity of living organisms and provides an insight into the importance of protein processing and structural modifications which are able to extend the functions of proteins [2]. Carbohydrate structures are post-translational modifications of proteins that are particularly important in multicellular organisms through their roles in structure and regulation. The structural complexity of the glycome far exceeds that of the genome as carbohydrate synthesis is not template driven (Figure 1). The difficulty in the analysis of glycan structure and function has resulted in the field of glycomics lagging behind that of the genome and proteome.

### 1.1. Structural Characteristics of Proteoglycans

Proteoglycans are widely distributed in animal tissues and are synthesised by nearly all types of cells [3]. Glycosaminglycans (GAGs), formerly known as mucopolysaccharides, are synthesized by covalent attachment to core proteins to form proteoglycans. The exception is hyaluronic acid (HA) which forms non-covalent linked complexes with proteoglycans. The covalent link between the core protein and GAG involves a specific tetrasaccharide linkage involving a glucuronic acid residue, two galactose (Gal) residues and a xylose (Xyl) residue. This structure couples to a serine (Ser) residue on the protein core structure via an *O*-glycosidic bond. In the case of keratan sulphate (KS) some forms are also linked via an *N*-asparaginyl bond. Proteoglycans are thus characterised by the vast diversity of GAG structures which decorate them. Members of the GAG family are long unbranched molecules which can be simplified into a repeating disaccharide structure, where one sugar is an uronic acid, either glucuronic acid (GlcA) or iduronic acid (IdoA) and the other is either an *N*-acetylated glucosamine acid (GlcNAc) residue or a *N*-acetylated galactosamine residue (GlcNAc). These sugar residues can be sulphated in different positions depending on the type of GAG. In the case of the simplest structural member of the family, hyaluronic acid, there is a repeat unit of GlcA and GlcNAc with no sulphate groups, which is in contrast to chondroitin sulphate (*D*-glucuronate and either GalNAc-4 or GalNAc-6 sulphate), keratan sulphate (Gal and GalNAc-6- sulphate) and dermatan sulphate (*L*-iduronate (which can be sulphated) and GalNAc-4- sulphate). Heparan sulphate (HS) is another type of GAG and is described in detail in the next paragraph. In general all GAGs are highly negatively charged which is essential for their functions which range from structural support in the matrix to lubricating joints, and regulating protein bioavailability and activity.

### 1.2. Heparan Sulphate Structure and Biosynthesis

HS is a ubiquitous linear polysaccharide molecule, belonging to the GAG family of macromolecules. HS chains are found covalently attached to core proteins to form HS proteoglycans (HSPG). Attachment of HS to different core proteins results in HS having the ability to alter its location and topography as core proteins such as syndecans and glypicans are targeted to the cell surface whereas perlecan, agrin and collagen XVIII are found in the extracellular matrix [4]. In addition, HSPG diversity is imparted by the multiple gene families of core proteins, such as four syndecan family members and six members of glypican family in mammalian cells [5]. Further, proteoglycan core proteins have spatial and temporal specific expression [6].

**Figure 2:**
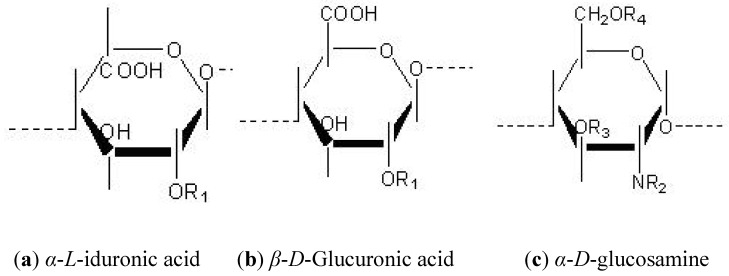
Individual monosaccharide units of heparan sulphate. The repeating dissacharide unit is composed of either α-L-iduronic acid (a) or glucuronic acid (GlcA) (b) and a N-acetylglucosamine residue (GlcNAc) (c). Various biosynthetic modifications can occur on the R positions of the monosaccharide units, involving functional groups, H- hydroxyl, COCH3- acetyl groups and SO3−sulphate groups. R1=H or SO3−, R2 =H2COCH3 or SO3−, R3= H or SO3−, R4= H or SO3−.

The expression of HS structure relies on a highly specific and regulated multistep biosynthetic process, which results in a vast array of possible HS structures. The possible modifications of HS structures can be simplified by viewing HS polysaccharides as a repeating disaccharide unit, composed of glucuronic acid (GlcA) and a *N*-acetylglucosamine residue (GlcNAc). The glucosamine may remain acetylated (GlcNAc) or be sulphated (GlcNS) at the amino position, or may also be found in an unsubstituted form (GlcNH_2_) [7]. The GlcA residue can undergo an epimerization reaction during biosynthesis and can also exist as *L*-iduronic acid (IdoA). The uronic acid undergoes 2-*O* sulphation, primarily on IdoA and rarely on GlcA. The glucosamine sugar can be further *O*-sulphated at the 6 or 3 positions. The monosaccharide units of these structures are depicted in Figure 2 [8].

### 1.3. Heterogeneity Supplied by the Domain Structure of HS Polysaccharides

HS disaccharide units form long chain polysaccharide structures. There are at least 8 different common disaccharide structures that make up HS. For longer oligosaccharides, the number of variant structures increases exponentially. For example, in the case of a hexasaccharide there are theoretically 512 (8^3^) possible structures and in the case of octasaccharides there are 4096 (8^4^) possible structures [9]. However, the constraints of the biosynthetic process limit the number of possible structural variations.

Though there is a vast amount of diversity in HS structures, there is also evidence of regulation in the formation and maintenance of these structures. This is evident from the specific domain-type structure that occurs in the full length HS polysaccharide chains, which typically vary between 50–200 disaccharide units long (that is equivalent to 25–100 kDa in size [8]. This manifests as regions of high sulphation, consisting of mainly IdoA residues sulphated at the 2 position (IdoA-2-S) and *N*-sulphated residues that are known as ‘S’ or ‘NS’ domains.

**Figure 3:**
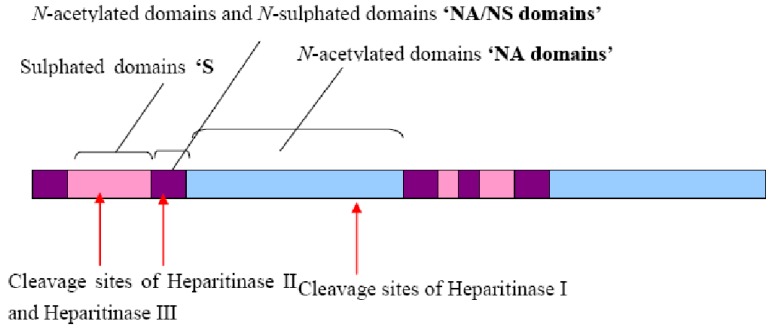
Diagram representing different domains present in HS chains. Sulphated domains (*S*-domains) contain *N*-sulphated disaccharides with IdoA-2-*O*- sulphate as a major uronate component. *N*-acetylated (NA) domains are non-sulphated and have acetylated regions. In contrast, *N*-acetylated and *N*-sulphated (NA/NS) domains contain alternating *N*-acetylated and *N-*sulphated units. The cleavage sites for commonly used heparitinase digestion enzymes are shown [11].

These domains are interspersed between unmodified domains, called ‘*NA*’ domains, which consist mainly of unmodified GlcA and GlcNAc residues. These two domains are separated with intermediate domains, which have an alternating arrangement of *N*-acetylated and *N*–sulphated disaccharide units. These are also known as “*NA/NS* domains” (Figure 3). The lengths of the *S*-domains are typically 3-8 disaccharide units whereas the lengths of the NA/NS domains are more variable [10].

### 1.4. Flexibility of HS Chains

Two factors control the flexibility of HS. One factor is the ring conformation and the other factor is the bending about the glycosidic linkage. The pyranose ring structure can have a chair (C), a skew shape (S) conformation, or a twist boat conformation (T) is also possible. Residues such as GlcN and GlcA are rigid and sTable 4C_1_ rings. But IdoA residues equilibrate between the ^1^C_4_ conformation and ^2^S_0_ conformations which are equi-energetic. This equilibrium is very fast (10^6^ second), under physiological conditions, so indicating that the energy barrier between the two forms is low. Modifications such as sulphation have effects upon neighbouring residues and may affect conformation equilibrium [12]. The second factor that affects the flexibility of HS is the rotation around the glycosidic linkages. This is a major consideration of three-dimensional structures, which depend on the bonds that form the glycosidic linkage of C1-O and O-C4 bonds. The rotations are represented by the dihedral angles, Ψ and Φ and rotation in these bonds result in these portions in the polymer being flexible [12].

### 1.5. Diverse Functions of HS

HS interacts with many molecules, particularly proteins, and has diverse functions. The length of HS chains allow them to span targets that can be located several hundreds of nanometers apart, such as a basement membrane or an adjacent cell. HS has many roles in cell physiology which can be broadly divided into different categories such as co-receptor functions with various growth factors (members of the FGF growth factors and Wnt signalling), involvement in cell adhesion, bacterial and viral attachment, organogenesis, embryogenesis and neuronal connectivity [13,14,15,16]. In addition specific HS structures have been shown to be responsible for specific functions. Specific sequences in HS structures have been shown to be responsible for specific interactions with proteins [1]. This was first shown with the pentasaccharide sequence in heparin binding to and increases the activity of antithrombin [17].

### 1.6. Variation in Endogenous Tissue HS Structures

The variation in HS can be analysed by extraction and purification of HS from tissues with consequent structural examination. These studies show that variation of HS structures occurs in a regulated manner. HS composition analysis of HS preparations from different bovine tissues [18] shows clear differences in HS structural compositions. The composition of *N*-sulfated regions in human organs shows differences in the occurrence of domains. For example the ratio of IdoA-aManR(6-OSO3) / GlcA-aManR(6-OSO_3_) decreases from 3.2 to 1.0 from healthy human spleens to diseased spleens [19]. There are differences in the proportion of the *O*-sulphated disaccharides, GIcA-aManR (6-OSO_3_), ldoA (2-OS0_3_) – aManR (6-OS0_3_), and ldoA – aManR (6-OS0_3_) from human cerebral brain compared to other organs such as liver, aorta and kidney; 2-*O*-sulphate GlcA units have been noted to be more abundant in adult cerebral brain but lacking in the neonatal cerebral cortex [20]. Other mammalian tissues have also shown tissue-specific structural compositions using disaccharide compositional analysis and oligosaccharide mapping, where lung contained high overall 2-*O*-sulphation levels and HS from spleen, kidney and lung contained high levels of 6-*O*-sulphation [21]. This indicates a strict regulation in the biosynthetic modification of polymers [13,22]. Further evidence for the regulation of HS structures is seen with immunohistochemistry analysis. Antibodies with different affinities and specificities for HS epitopes show that HS structure and distribution varies in tissues [23,24]. Further, different staining patterns demonstrate that distinct cellular compartments in tissues possess different HS structures [25,26]. Differences in the structural composition of HS also exist with tissues of different ages where elevated levels of the trisulphated disaccharide structure are found in older subjects [27].

## 2. Structural Analysis of HS Glycan Chains

The most common method of determining differences in HS from different sources is the analysis of disaccharides. The disaccharide composition of HS can be determined by calculating the proportion of each identified disaccharide structures. Although the same disaccharides may be present in different tissues, the proportion of each disaccharide may vary quantitatively resulting in structural differences between tissues. The domain structures of HS can also be analysed to understand the relative distributions of NA domains and the variable length of the NS and NA/NS domains [28]. The structures found in different domains can also be analysed by oligosaccharide mapping which provides a ‘fingerprint’ of the structure [29,30,31]. Sequencing has also been used to provide more detailed information on the structure of individual oligosaccharides [32].

### 2.1. Indirect Methods To Study HS Structure

The hydrophilic nature of HS coupled with their lack of a suitable chromophore mean that direct analysis of HS structure is not possible. Originally depolymerisation of HS was achieved using nitrous acid hydrolysis. Nitrous acid cleaves in between the hexosamine residues and the hexuronate residues, where the amino group is either *N*-sulphated or unsubstituted. This results in the conversion of the *D*-glucosamine at the reducing-end of the structure into a 2,5- anhydro-*D*-mannose residue and the hexuronate residues are left intact [33]. This reaction has been extensively investigated and shown to be pH dependent. In low pH conditions such as below 2.5, the direct deamination of *N*-sulphated glucosamine residues is possible [34]. However at pH 4, *N*-unsubstituted residues are deaminated more rapidly.

Disadvantages in employing nitrous acid digestion is the lack of a chromophore on the resulting oligosaccharides, which means chemical digestion and separation of such fragments can only be followed at low sensitivity [8]. For this reason, this technique is combined with reducing-end labelling techniques such as radiolabelling, hydrophobic tags or fluorescent tags. The labelling reaction of the reducing-end of nitrous acid treated fragments is aided by the fact that the 2,5- anhydro-D-mannose residue produced at the reducing-end is highly reactive.

Another widely used method of degradation of HS involves enzymatic digestion. There are two types of enzymes capable of degrading HS structures and include the polysaccharide lyases (derived from prokaryotic sources) and the other being glucuronidases (hydrolases also known as heparanases) derived from eukaryotic sources. The former heparinases (also known as heparitinases) are derived from *Flavobacterium* and cleave HS molecules between the C4 and C5 carbon of iduronic acid or glucuronic acid, via an elimination reaction [35]. This introduces an unsaturated double bond at the uronic acid residue at the non-reducing end. This double bond aids detection of the resulting digestion products as it strongly absorbs in the UV region of light with a maximum absorbance at 232 nm and a molar extinction coefficient of 5500 M^−1^cm^−1^ [36]. However, the disadvantage of this method is that the original identity of the uronic acid is eliminated. These enzymes have now been characterized and though they have 15% sequence alignment [37] these enzymes have distinct specificities as listed in (Table 1). These enzymes provide useful tools for structural analysis of HS: when used singly or in combination, for varying times, they can produce different degrees of digestion in certain domains of the HS polysaccharide chain.1.9. Studying the Domain Structure of HS

### 2.2. Studying the Domain Structure of HS

Enzymes such as heparitinases are a vital tool used in the study of HS structure. Complete digestion of HS polysaccharides into disaccharide structures can be achieved using a cocktail of enzymes (heparitinases I, II and III). The enzymes can also be used individually to selectively cleave regions of HS. The resistance of certain parts of the oligosaccharide chains to enzyme activity means that selective analysis can be carried out in the different domains. Portions of the HS structure can also be treated with highly sensitive exolytic lysosomal enzymes, which act at the reducing terminus of saccharides provided that they have suitable substrates [39]. A brief summary of all the enzymes available and the specificities is given in Table 1. For example a sequence containing 2-*O*-sulphate could be detected by susceptibility to iduronate-2-sulphatase (I2Sase). These enzymes are used in combination with electrophoretic techniques (integral glycan sequencing) as well as mass spectrometry techniques.

### 2.3. Study of HS from Tissues

HS is omnipresent in all phyla and is considered as evolutionarily ‘old’ [14]. Methods of extraction and purification of other types of polysaccharides are not suitable for isolation of HS. The isolation of HS has once been described as: ‘finding the needle in the haystack by the arduous task means of removing every single blade of hay and hoping the needle will remain evident’ [40]. Many studies have extracted and purified HS from a range of mammal tissues. More unusual sources have included unossified antler of *Cervus elaphus* [41], but more commonly, laboratory animal models have used including mice [21] and rats [42]. Larger animals used, particularly for commercial purposes, have been pigs and cows [43]. However, there are limitations to the traditional extraction and purification methods, which mean that smaller quantities of endogenous tissue, such as embryonic tissue and subsections of tissues, cannot be analysed using these procedures. In order to overcome these problems indirect methods have been devised. This involves transforming tissue into cultured cells [44]. Valuable structural information has been obtained in this way. However, structural differences occur between immortalized culture cells and endogenous tissue [45]. Studies also report structural changes at different stages of cell culture [46,47]. Therefore, in order to avoid problems associated with *in vitro* cultured cells, an extraction and purification procedures are essential to directly characterize small quantities of endogenous tissue and compile reliable *in vivo* structural information (see Section 2.6).

**Table 1:** Exoglycosidases and exosulphatases for HS structural analysis. Sulphatases remove sulphate groups from the non-reducing end (NRE) and glycosidases cleave the whole non-sulphated monosaccharides. Information as taken from [38].

Enzyme	Substrate Specificity
*Bacterial Exoenzyme*	
Δ4,5-glycuronate-2-sulphatase	ΔUA(2S)
Δ4,5-glycuronidase	ΔUA
*Exoglycosidases*	
Iduronidase	IdoA
Glucuronidase	GlcA
α- N-acetylglucaminidase	GlcNAc
*Exosulphatases*	
Iduronate-2-sulphatase	IdoA(2S)
Glucosamine-6-sulphatase	GlcNAc(6S). GlcNS (6S)
Sulphaminidase (glucosamine *N* –sulphatase)	GlcNS
Glucuronate -2-sulphatase	GlcA(2S)
Glucosamine -3-sulphatase	GlcNS(3S)

### 2.4. Traditional Methods for Extraction and Purification of HS

In general, purification of proteoglycans can be achieved by common molecular purification using procedures which select distinct chemical properties of a particular class of glycosylated proteins. In particular, HS proteoglycans have a high specific gravity and a high molecular density. This occurs from the high negative charges of the sulphate and carboxyl groups present [48]. Traditionally methods use dialysis with high concentrations of harsh organic solvents such as 8 M urea and addition of trichloroacetic acid [49] or guandinium chloride [50]. These substances are chaotrophic agents and disrupt the three dimensional structures of proteins, DNA and RNA. Urea and other chaotrophic agents achieve this by interfering with the stabilizing inter-molecular interactions which mediate non-covalent forces such as hydrogen bonds, van der Waals forces and hydrophobic effects. The disadvantages of these older methods include the fact that they are lengthy: can involve multiple sample transfers, and involve harsh chemicals (could alter pH and cause de-*N*-sulphation [51]. Other methods include *β*-elimination, which involves alkaline borohydride treatment, which acts to disrupt the serine-xyloside linkage which attaches GAG chains to the core protein backbone. This results in the release of intact HS from protein cores [52]. Another method of HS purification from cells is using non-ionic detergent solution, such as 1% Triton X-100 0.5 M KCl, which can then be subjected to density centrifugation in caesium chloride (CsCl). This causes the separation of molecules, according to their density [53]. The degree of hydrophobicity can also be used in order to separate different structures of HSPG. The degree of hydrophobicity can be used to separate GPI-anchored HSPGs from those which contain transmembrane regions using hydrophobic chromatography such as Octyl-Sepharose resin [54]. HSPGs containing GPI-linkages bind with high affinity to Octyl-Sepharose while low molecular weight HS chains bind with weaker affinity to Octyl-Sepharose. Electrophoretic techniques can be used for separation of proteoglycans. This technique can either be used separately or in combination depending on the degree of purification required or type of HSPG to be purified.

### 2.5. Structural Analysis of Cellular and Tissue Derived HS

HS does have a natural chromophore, the acetyl group which absorbs at 215 nm. However, this is not very strong and the low UV is not convenient as many other molecules also absorb at this wavelength. Therefore a more convenient method of detection involves introducing a chromophore. This is made possible by using digestion with heparitinase enzymes which introduces a double bond. Separation and detection can be undertaken using a range of techniques. In the past the use of radiolabelling has been important in studying HS from different cell culture and tissue sources. One method of radiolabelling used ^35^*S*, which incorporated into newly synthesized HSPGs in conjunction with pulse-chase experiments to study HS structure and turnover [55]. Other types of radiolabelling include the incorporation of [*^3^H*] glucosamine with detection during anion exchange HPLC methods [47,56]. However, radioactivity poses health hazards and increased complications in the disposal of waste.

Direct UV measurements are generally preferred, which result from the digestion of heparitinase enzyme digestion as previously *described.* UV analysis has been used extensively to detect HS from different sources from unossified tissue [57] to characterisation of HS from bovine organs using CE detection [58]. However this detection method suffers from low sensitivity.

New methods of HS detection from smaller quantities of tissue have been developed using fluorescent molecules conjugated to the reducing end of free HS disaccharides and oligosaccharides. Detection is facilitated by the use of standard fluorimetry methods or laser-induced fluorescence detectors for increased sensitivity. Different fluorescent tags can be used depending on the wavelengths, stability and sensitivity required.

### 2.6. Fluorescent Tags Which Offer Higher Sensitivity

The sensitivity of detection can be increased by attaching different fluorescence tags to the reducing end of heparitinase digested HS disaccharide structures. Different conditions can be achieved by using different fluorescent tags. Examples of fluorescent tags are: amines, hydrazides and hydroxyl amines (also known as amino-oxy). Fluorescent tags have a lone-pair of electrons which facilitates nucleophilic attack of carbohydrate (anomeric) carbonyl group. Fluorophores which have been coupled to HS disaccharide structures include 2-aminobenzamide (2-AB) [59,60], 2-aminobenzoic acid [32,61] and also fluorescent aniline tags [62]. These tags together with 2-cyanoacetamide have been used in combination with reverse phase separation to achieve characterization of HS from embryonic stem cells [63]. Picogram levels of detection have been reported [64]. However, the disadvantage of these fluorescence tags is the requirement of additional paper chromatography methods to separate unreacted tag from saccharides. One promising and flexible tag is 2-aminoacridone (AMAC), which has been used to tag HS disaccharides. It is compatible with SAX-HPLC and CE separation and gives 10 times more sensitivity than UV detection [65,66]. Another promising fluorescent tag is BODIPY^®^ FL hydrazide which has been shown to label heparin and HS disaccharide structures [67,68]. The advantage of using BODIPY is the presence of hydrazide groups, which improve reaction yields. The extinction coefficient of this fluorophore is also significantly higher than previously used fluorescent tags (*ε* = 71,000 M^−1^ cm^−1^). The excitation of the fluorophore is 503 nm which is closer to the wavelength of common lasers (488 nm) than other fluorescent tags (AMAC is 425 nm). BODIPY^®^ FL hydrazide can be reversibly coupled to the reducing-end of the aldehyde group in HS saccharides to form a Schiff’s base which when reduced with sodium borohydride can form a stable amine derivative; it has femtomolar detection sensitivity [67,69].

### 2.7. Use of Separations Techniques for Structural Analysis

There are a number of different ways of analysing HS structure, which depend upon different factors such as the source and quantity of materials, the type of data which is required and the preparation and/or treatment of HS prior to the analysis process.

NMR spectroscopy and mass spectrometry are available (for examples, see [65] and have been able to provide direct sequence information. However NMR required relatively large quantities of materials (millimolar to micromolar quantities) and both require specialized instruments and techniques which are not routinely available. This leaves chromatographic techniques as the most common way to examine HS structure.

#### 2.7.1. Strong Anion Exchange Chromatography

In general, routine separation of HS relies upon gel chromatography and high-performance liquid chromatography (HPLC). Oligosaccharide products can be purified and separated according to their differing molecular weight measurements, other properties such as mass, charge and hydrodynamic volume. Different methods can be used for detection: **radioactivity measurement** can be used to detect ^35^S and ^3^H in samples prepared by biosynthetic radiolabelling (e.g., cultured cells) or where oligosaccharides (digested using nitrous acid) are labelled at their reducing-ends with ^3^H–borohydride; **UV detection at 232 nm** to detect lyase digested products. Other UV wavelengths can also be used such as 215 nm, which detects acetyl bonds, although this is less sensitive than detection of lyase products; **fluorescence detection** to detect fluorescently-tagged oligosaccharides. Strong anion exchange chromatography techniques are used for the separation of structures according to their negative charge. A common column is the Propac PA1, which is made of quaternary ammonium functional groups attached to nonporous core particles by agglomerated MicroBead™ resin. This strong anion exchange column enables the effective separation of disaccharide and oligosaccharide HS structures. Other benefits of this column are its stability and the column remains positively charged over a wide range of pH values [70]. SAX-chromatography can be used in combination with PAGE separation, for the purification of HS oligosaccharides [71]. Alternatively to SAX chromatography, hydrophilic interaction chromatography (HILIC) separates oligosaccharides on the basis of their overall polarity [72]. HILIC combined with MS has already been used for the separation and analysis of glycoproteins and GAG oligosaccharides [73,74].

#### 2.7.2. Polyacrylamide Gel Separation

The separation of HS structures is also possible using polyacrylamide gel electrophoresis (PAGE) which is routinely used for the separation of different sized oligosaccharide structures. Proteoglycan separation, using PAGE was first shown in seventies with composite gels made from 0.6% agarose and 1.2% acrylamide [75]. However, PAGE can also be used without agarose [76] and at higher percentages such as 33% with 19:1 acrylamide: bisacrylamide ratio. These gel compositions allow a great improvement in resolution than the standard SDS-PAGE gels where separation is hampered by the presence of the negatively charged side chains. This would result in smears due to structural heterogeneity of HS. Proteoglycans derived from cartilage extracts were separated in this way [77]. The use of fluorescent tags to label digestion products at their free reducing-ends via reductive amination reaction has also been determined. The lyase-digested products can be stoichiometrically coupled to a fluorescent tag and separated using high percentage gels. Picomolar concentrations can be viewed using a transilluminator.

#### 2.7.3. Capillary Electrophoresis

A further extension to electrophoretic separation of HS is capillary electrophoresis. Capillary electrophoresis (CE) was developed in order to combat the two main drawbacks of conventional polyacrylamide gel electrophoresis. Firstly, the problem of resolution, which was caused by the low voltages achieved from the ‘Joule heating’ effect. This is encountered in CE; a fine capillary is used, which allows a high surface to volume ratio and efficient cooling. Secondly, conventional electrophoresis results in a low level of detection due to indirect detection using dyes. Using CE, it is possible to directly detect molecules using a narrow window in the capillary. Detection uses UV for bacterial lyase digested HS [78] or by using laser induced fluorescence detection module and pre-derivatization of the HS (by using different dyes as explained in section 2.6.). Additional advantages of CE are the efficient and fast analysis (due to short run times). CE is based on the separation of molecules using the electrophoresis process in free solution or in a capillary filled with a soluble matrix. Separation of HS and heparin is achieved using free solution, also called “capillary zone electrophoresis” of CZE [79].

#### 2.7.4. Integral Glycan Sequencing (IGS) Techniques

Separation using gel electrophoresis in combination with exolytic lysosomal enzymes has led to techniques that directly determine structural information. This was originally described by Turnbull *et al.* [32] as a strategy called ‘integral glycan sequencing’ or IGS. This method involves labelling the reducing-end of an oligosaccharide via a reductive amination step using the fluorescent tag 2-aminoacridone (2-AA) in special formamide reaction conditions. Following a rapid gel filtration step, to separate tagged saccharide from unreacted tag, the fluorescently labeled products can be further digested with partial nitrous acid digestion. This forms a ladder of evenly numbered oligosaccharides (di-, tetra-, hexa-, *etc.*). These can then be digested with specific exoglycosidases and exosulphatases. These enzymes result in the selective removal of sulphate or monosaccharide units from the non-reducing end of the oligosaccharide ladder products. Similar methods have been used with radiolabelled substrates followed by SAX-HPLC separation [80,81].

Structures are then separated using high-density polyacrylamide gels with fluorescence visualization of the products. Changes in banding positions from different enzymatic treatments are then compared and sequence data can be directly interpreted from the band shifts [82]. The sensitivity of this technique allows the detection of nanomolar quantities of HS. Improvements have also been shown using an alternative label of 7-aminonapthalene-1,3-disulfonic acid, with advantages of increased sensitivity levels [39].

### 2.8. The Use of Mass Spectrometry for Sequence Information

Mass spectrometry has become a critical technique in determining the sequence information of HS structures. The overall negative charge of HS means that structures are difficult to ‘fly’ using conventional ionization methods. Additional caveats are the fragility of structures and the ease of fragmentation. Therefore, only two main types of soft ionization techniques are used in HS sequence analysis. These techniques are: matrix-assisted laser desorption/ionization which are used in combination with time of flight detection (MALDI-TOF); and electrospray ionization (ESI).

Analysis of HS with MALDI uses complexation with a positively charged peptide. The positively charged peptide binds to the negative charges of HS and masks these charges and allowing ionization. Detection therefore from the mass analysis of this combined structure. Property-encoded nomenclature in conjunction with MALDI (PEN-MALDI) [83] was used to sequence the decasaccharide structure of the ATIII binding site from heparin [84] in combination with IGS and 1 dimensional, 1D proton NMR [85].

MALDI-TOF suffers from resolution problems which arise from the uronic acid epimers or the position of the *O*-sulphates on the C3 or C6 position of glucosamine and also the use of MALDI suffers from dissociation of sulfate groups that occurs as a result of the ionization process [86]. ESI-MS has advantages over MALDI-TOF and relies on masking negative charges by ion pairing using positively charged quaternary ammonium ions. This is a volatile substance and can be evaporated. This enables direct mass calculation of HS fragmentation. It also improves peak retention time and shape [87]. The ESI technique offers solutions to the problem of distinguishing isomers, by using tandem MS/MS which can detect differing fragmentation patterns [88,89,90,91]. Alternatively, IGS can be used in conjunction with ESI [92]. Additional advantages of ESI are that direct analysis is possible using reverse phase separation chromatography [92,93], graphitized carbon LC-ESMS [94] or capillary electrophoresis equipment to separate structures prior to ionization. Increased sensitivity can also be achieved using nano-spray ESI [95] Negative polarity ESI-MS is often used to detect HS saccharides as deprotonated ions. This method has been used for on-line LC/MS detection of HS saccharides separated using hydrophilic interaction chromatography and size exclusion chromatography. Positive polarity ESI-MS may be used when the saccharides are paired with quaternary ammonium ions to give a net positive charge. The ion pairs enable retention of HS saccharides using reversed phase chromatography [96].

## 3. Current Methods for Analysis of Large Data Sets Obtained from Structural Studies

Spectroscopic data analysis gives rise to spectrum of peaks which results from the differential separation of structures. Peaks have a certain retention time, height and peak area depending on the sample and separation method. The peak area is measured by the electronic integration of resonance signals in a spectrum [97]. However many difficulties are experienced when using strong anion exchange chromatography for oligosaccharide structural analysis of multiple data sets. These are difficulties in drawing different spectrums on one chart without graphs overlapping at different points (hence avoiding ‘noise’ and obscuring regions of the spectrum), difficulties in obtaining quantitative observations, and drawing conclusions from the data sets

Most instruments are now computerised and automated. This facilitates the collection of large volumes of data, and aids the simultaneous monitoring of different experimental parameters. However, there are still problems in comparing data and drawing conclusions. Therefore, chemometric data analysis is needed to solve these problems with comparative data analysis and can be used to extract information from the different data sets.

### 3.1. The Need for Chemometric Techniques for HS Structural Analysis

The complex structure of HS polysaccharides can be simplified into a repeating disaccharide unit which is modified into 8 commonly occurring disaccharides. However, the study of HS oligosaccharide structures is more complex. This is because longer HS chains have exponentially greater numbers of theoretical structures. A decasaccharide structure can have a possible 32,768 (8^5^) theoretical structures [98]. Experimentally, far fewer structures are detected [99]. The techniques used in the study of HS oligosaccharide structures are often spectroscopic techniques such as NMR or analytical chromatography, which separate on the basis of size using gel-filtration, or charge using strong anion exchange chromatography. Common techniques used for HS sequence determination are mass spectrometry techniques such as MALDI-TOF or ESI-MS/MS. All these techniques share the following characteristics which make comparative analysis very difficult:

(1). The sensitivity of the methods means they are good at sample analysis, especially when comparing with standards (if possible). But, when standards are not available (which is common in HS oligosaccharide analysis), large differences in spectral results are caused from small changes in sample processing and handling. This makes comparison of results very difficult.
(2). The high dimensionality of the data from experimental techniques results in ‘noisy’ spectra (white noise) which may require pre-processing. This is a challenge as one has to be careful not to remove significant components.
(3). One of the most common problems for all techniques that study HS oligosaccharides is that simultaneous and routine analysis provides a vast quantity of data.

### 3.2. Existing Chemometric Methods

Other fields of biological research have experienced handling large volumes of data on a routine basis, and standard software packages have been developed to overcome this problem, such as microarray analysis. This uses a combination of chemometric or statistical analysis to deal with data sets. The glycomics field has somewhat lagged behind in this area and has only started to use chemometric methods in recent years. Multivariate chemometric techniques employ mathematics to compute matrix algebra and vectors and can be divided into two important applications [97]. Another example of pattern recognition is cluster analysis. Cluster analysis has already been used to simplify relationships relating to HS structural and functional analysis. Cluster analysis ascribes objects into groups (called *clusters*) and partition objects into the same cluster if data is considered as ‘similar’. If data is considered as different, then data is clustered into separate groups. Similarity is determined by the type of distance measurement which is routinely measured by statistical data analysis. Cluster analysis has been successfully used to compare similarities in spectra of synchrotron radiation circular dichroism (SRCD) [100]. There are different types of clustering techniques such as hierarchy clustering, *K*-clustering and self-organising maps. One way to represent cluster analysis results is to produce a cluster heat map which is a high-resolution colour graph that uses colour representation to depict another dimension of data. Heat maps are typically used in molecular biology techniques such as DNA microarrays which represent the levels of expression of many genes across a number of comparable samples. New tools have also been developed to aid in the analysis data deriving from MALDI and ESI-MS [101]. In addition software has been created to profile glycans present in samples separated and analysed using liquid chromatography/mass spectrometry (LC/MS). An example is the GlycReSoft software package, created to allow the automated recognition of glycans from LC/MS data [102].

### 3.3. Databases Crucial in the Intercalation of Data

Many internet tools are also available which provide access to software that extract information from experimental research [103]. In Europe, the main website which collates all this information is EuroCarb-DB. The software available has various functions, such as predicting potential glycosylation sites on proteins, predicting the three-dimensional structures of carbohydrates as well as profiling different HS structures [103,104,105]. New tools have also been developed to define the reproducibility of samples, through the use of principles from the chaos theory [106].

## 4. Functional Analysis of HS Structues

There are many ways to study the biological function of HS structures in order to understand the structure-activity relationships (SAR). In general, the sources of the structures are from:

-Direct extraction and purification of HS populations from tissues or culture cells arising from different organs.
-Partial chemical and enzymatic modifications of heparin to produce oligosaccharides of controlled modifications and size [98]. The structure of these fragments can be confirmed using disaccharide compositional analysis as well as sequencing methods (Section 2).
-Chemically synthesized HS oligosaccharides [107]. Synthesis of HS structures is a lengthy and specialized process. Solid-phase synthesis and combinatorial chemistry has greatly advanced this field [108,109,110]

### 4.1. Techniques for Studying Structural Aspects of HS and Protein Interactions

Crystallographic studies have been very important in gaining insights into HS/protein interactions, as previously seen with FGF-2. Techniques which immobilize HSPGs onto surfaces have been very important in understanding binding kinetics and affinities of HS with different binding partners. Biosensors have been used to study many HSPG interactions. More recently, optical and acoustic techniques such as dual polarization interferometry (DPI) [111], quartz crystal microbalances [112], synchrotron radiation circular dichroism (SRCD) [113] and Fourier transform infrared spectroscopy (FTIR), have been described to allow the further understanding and quantification of the molecular dynamics of HS interactions [111]. The immobilization of non-derivatized oligosaccharides onto microarray surfaces has also allowed qualitative and quantitative analysis of protein binding interactions through fluorescence detection, and allows the simultaneous analysis of a large number of samples [114,115,116].

### 4.2. Investigating Effects on Cellular Signalling

The importance of HS as a co-factor in FGF signalling was first shown in cells that lacked HS naturally or where sulphation of HS chains is blocked using sodium chlorate treatment [117,118,119]. Further study into the relationship between HS structure and function was carried out by screening size-defined or chemically desulphated heparin saccharides for their ability to activate FGF signalling. Saccharide structures were found to be specific in their activity since sulphation of particular residues (such as 6-*O*-sulphation of glucosamine) is essential in FGF-2 signalling using FGFR-1 [120,121]. A regulatory role of HS has also been highlighted in experimental work which shows that selected structures are capable of activating and inhibiting specific FGF/FGFR isoform combinations [32,99].

### 4.3. Cellular Signalling Assays

Research into HS ultimately aims to elucidate the structures responsible for different functional outcomes. Therefore information from biophysical assays and crystallography data are combined with information from cellular assays. Different types of cellular assays are used to determine the effect of specific oligosaccharide structures and the choice is dependent on cell type, sensitivity of the assay, material required, detection equipment available and the type of methods involved. There are four main types of cellular assays including: Cell proliferation/ DNA synthesis; cell adhesion, aggregation and invasion assays; angiogenesis assay; cellular signaling.

#### 4.3.1. Cell Proliferation

The ability of HSPGs to bind to different FGF growth factors and growth factor receptors to induce DNA synthesis or cell proliferation has been extensively studied [122,123]. Cells naturally deficient in HS such as BAF cells provide an important tool for the study of the functional effects of different HS structures, different FGF and different FGFRs [124]. Cells can also be treated to remove sulphation of HS. This is achieved by addition of a competitive inhibitor such as sodium chlorate which inhibits sulphation process [117]. In normal conditions sulphate is incorporated into HS using a sulphate donor adenosine 3'phosphate 5'-phosphosulfate (PAPs), which is then transported to the Golgi apparatus, where sulphate groups are then transferred from PAPs onto mature GAG chains. Though PAPs is a obligate sulphate source for cytosolic and membrane-bound sulphotransferases, 70% PAPS consumption is by GAG biosynthesis [125]. An effective method for removing sulphation and allowing abrogation of FGF growth factor induced signalling and so different combinations of FGF growth factors and receptors is by sodium chlorate inhibition. Radioactivity can be used to measure DNA synthesis by measuring the incorporation of *methyl*-[3H] thymidine. Cells numbers can be quantified using colorimetric products such as 3-(4,5-Dimethylthiazol-2-yl)-2,5- diphenyltetrazolium bromide, a tetrazole (MTT) which is reduced by the mitochrondria of living cells [9].

#### 4.3.2. Cell Adhesion and Invasion Assays

Cell adhesion assays have been important in assessing the ability of cells to adhere to a surface. Cells can be suspended in a buffer and allowed to interact by gravity. The number of adhered cells can be ascertained with a haemocytometer or a Coulter counter to count the number of cells. Cell invasion assays have also been useful in assessing the ability of cells not only to adhere but also enter cells, such as myeloma cells. A hydrated type I collagen gel can be used to assess the distance and depth of cell invasion. Detection using phase microscopy has been useful in measuring the depth of cell invasion. Non-specific interactions can also be removed by protease digestion of non-invasive cells. These assays can provide information on a number of factors as they are easily manipulated using promoters or inhibitors [126].

#### 4.3.3. Angiogenesis Assays

HSPGs are involved in the sprouting of new blood vessels from the existing microvascular system (angiogenesis) as they bind many inhibitors of this process [127]. Angiogenesis assays can be used to assess the involvement of many different proteins. Many angiogenesis kits are now commercially available but the original assay was described over 50 years ago, using chorioallantoic membrane (CAM). Fertilised chicken eggs can be used which have a small window in the shell which exposes the CAM and filter discs pre-soaked with growth factor and HSPG can be added. The window is sealed and incubated and the grafts are scored for growth and vascularization [128]. Assays with cultured endothelial cells can also be employed to examine vessel formation [129].

#### 4.3.4. Cellular Signalling Assays

A common method to study the downstream effects of different structures of HSPGs is by detection of different proteins and protein phosphorylation involved in different cellular signalling pathways [130]. This is commonly carried out using western blot analysis but more recently has involved immunostaining techniques. The problem with the assays mentioned is their limitation in the number of samples which can be analyzed. The assays tend to use microtitre plates in 96-well and less commonly a 384-well format. However manual preparation has to be considered such as human error associated with sample preparation which can be difficult and time consuming.

### 4.4. The Need for Higher Throughput Biological Assays for the Study of HS

Higher throughput assays are now being favoured for the study of many molecules. The use of higher throughput (HTP) assays in the study of HS would improve the study of HS as difficulties in purifying HS mean that only small quantities of certain structures are available. Using a HTP assay means: reduced time for experiments; elimination of radioactive waste and reduction in chemical waste; reduction in quantities of HS saccharide and valuable consumables needed.

### 4.5. Use of Cell Based Screening Techniques

Traditional cell based screening techniques using microtitre plates suffer from low numbers of samples which can be studied simultaneously. The sequencing of genomes has drastically changed the emphasis and direction of research. In this post-genome era emphasis has been put on validating specific gene targets with particular functions and disease states, and has led to biopharmaceutical companies increasing their cell based screening activities from 30% to 50% [131]. The use of microarrays, in place of other assays, has been successful in studying gene expression and protein interactions. The automation and high-throughput nature of microarrays has allowed rapid advancements in functional understanding of genes and proteins.

### 4.6. Glycoarrays-Carbohydrate Microarrays

DNA microarray platforms were first described and patented in 1998 for the analysis of the expression of different genes and quantitation of nucleic acid and protein interactions [132]. Microarray platforms have now been modified to develop novel platforms, such as protein arrays which study molecular interactions of proteins. More recent platforms such as cell array or reverse transfection arrays have been described [133]. Cell arrays consist of plasmids or RNA spotted on microarray slides and co-cultured with overlaid cells for transfection. Successful transfection is detected by an increase in fluorescence corresponding to the construct used and localization on the slide. These techniques allow high-throughput parallel screening and have been championed as a strategy for large-scale experiments such as genome-wide RNAi screens [134] and in screens for new genes or drugs [135,136]. Cell arrays have also been used to screen the effect of overexpression of human genes on cell apoptosis [137].

Many different types of cell array platforms have emerged to date. Tissue arrays contain arrays of cylindrical tissue biopsies from individual donor paraffin embedded tumour blocks which are placed in a certain position onto a ‘recipient’ paraffin block, using specialist equipment. Tissue arrays allow the parallel detection of DNA using fluorescence *in situ* hybridization (FISH), RNA using mRNA *in situ* hybridization and proteins using immunohistochemistry. Tissue arrays allow the simultaneous analysis of 1000 tissue biopsies [138] and have important clinical applications in cancer as they can potentially analyse large numbers of tumour tissues and can be used to study diseased and normal tissues in humans, as well as transgenic tissue in mice [138].

The use of microarrays for the study of carbohydrate interaction (also known as **glycoarrays** or **glycan arrays**) has lagged behind other biological molecules, with scientific literature appearing only in 2003 [2]. The functional study of the heterogeneous structures of HS has always proved problematic because of the limitation in the relatively low number of samples which can be simultaneous analysed in microtitre plates. Glycan microarrays offer the possibility of high-throughput analysis of carbohydrate structures.

### 4.7. Fabrication of Carbohydrate Microarrays

The construction of a carbohydrate microarray requires more technical considerations than other biological molecules. One of the key components of a glycan array is the surface. Typical surfaces are microtitre plates, functionalised glass slides, nitrocellulose coated slides and gold slides [139]. The choice of surface is important as it determines the type of detection which can be used, as the detection method depends on the fundamental properties of the surface.

The immobilization of the glycan structures onto a surface is the second consideration. There are four main chemistries for the attachment of carbohydrates to surfaces which are listed below:

-Reducing-end aldehyde linked to amino and hydrazide surfaces groups on the surface.
-Immobilization using Diels-Alder reaction.
-SH-/malemide.
-Site specific and non-covalent immobilization.

Using the reducing-end aldehyde method linked to amino or hydrazide derived surfaces it is possible to link all carbohydrate structures containing a free aldehyde groups at the reducing end of the sugars. Enzymatically digested oligosaccharides and disaccharides of HS, CS, DS and KS can be immobilised [140,141]. Disadvantages of using hydrazide-derivatized surfaces of gold is that a spacer is needed between the surface and glycan to increase accessibility of the glycan chain in solution, such as a ω-thiolated alkyl chain (C16) self-assembled monolayer [115]. The advantages of gold are a variety of detection methods can be used such as matrix- assisted laser desorption/ionization time-of-flight (MALDI-MS), surface plasmon resonance (SRP) biosensors and quartz crystal microbalances [115]. Immobilization using the Diels-Alder reaction has been shown for immobilization of cyclopentadiene cyclopentadiene-containing carbohydrates on a benzoquinone-coated gold surface and characterized by Mrksich and Houseman [142]. The advantage of this method is that the reaction is efficient. Malemide-linked sugars attached to thioether linkages to slides coated with thiol groups have been described [143]. The disadvantage of this technique is that steric hindrances of proteins binding to carbohydrates need to be considered. So tethers of proper lengths need to be inserted between the maleimide groups and the carbohydrate moieties. The use of site-specifically and non-covalently conjugating carbohydrates onto unmodified surfaces have been described for neoglycolipids (NGLs) using nitrocellulose [144,145] Advantages are no previous surface modifications are needed which reduces experimental time.

One other method of attaching carbohydrates involves the passive adsorption of structures onto the surface. This surface uses black polystyrene slides and a physical surface modification of MaxiSorb™. The binding of carbohydrates onto the surface involves hydrogen binding, ionic binding (involving polar parts of the molecules) and van de Waals hydrophobic interactions. Immobilization is strengthened by the removal of water between the immobilised carbohydrate and the hydrophobic surface [146].

### 4.8. Glycobioarrays

Current *in vitro* functional assays using cell biology approaches often require high cell numbers or larger amounts of reagents that are not always available, especially in the case of saccharides. Therefore, the development of slide-based functional screening assays may allow the screening and differentiation of different and larger populations of saccharide structures. Although array methods have been described employing living cells for RNAi transfection and small molecule responses [135,136,147,148], there have been few research studies which measure binding of bacteria, viruses, and mammalian cells to immobilized glycans [149,150,151,152,153]. An example of a glycan array used for the quantitative assessment of cell adhesion has been shown using glycan arrays with lectin structures on hepatocytes [152]. This has been shown using glass slides with covalently attached monosaccharides and oligosaccharides of non-reducing terminal *N*-acetylgalactosamine (GlcNAc)residues, galactose (Gal) and *N*-acetylgalactosamine residues. Primary chicken hepatocytes express a well-defined *C*-type lectin that binds to non-reducing terminal *N*-acetylglucosamine residues, and was labelled with a fluorescent dye. A specific chamber was used to remove non-adherent cells (GlycoChip® Centrifugation Chamber) and adherent cells were measured using fluorescence detection. Chicken hepatocytes bound selectively to lectin derivatized with GlcNAc structures rather than spots of lectin with Gal or no modifications [152].

However a more recent study shows that a slide-based technology utilizes the immobilization of saccharides onto aminosilane surfaces via a Schiff’s base linkage with the reducing ends in order to assess cellular reponses to glycans [154]. Significant potential for further development as a generic tool for functional glycomics screening of a wide range of glycans is shown with this tool [154]. The methodology is easily applicable to a standard laboratory setting because images are acquired using only a DNA microarray scanner. It has a number of advantages, including rapidity, provision of miniaturization (and thus low reagent usage), and also significant potential for development as a high-throughput tool.

## 5. Concluding Remarks

Glycomics techniques for the large scale structural and functional analysis of HS glycans now have real potential to be extended to study natural HS saccharide libraries, the production of which has recently been described [155], and also HS saccharides produced by synthetic chemistry [156,157,158]. Furthermore, such saccharides can be robotically spotted into array formats [116,156,157,159,160] that would allow the large-scale simultaneous analysis of different structures. The development of array-based assay technologies is a much-needed advance for assessing glycan function, especially in the case of the HS proteoglycan family. Such studies could subsequently address the structure-function relationships of HS saccharides at a functional level, and might be further exploited as a generic tool for glycan analysis. Ultimately one can readily envisage that glycomics studies of HS will yield new insights which underpin opportunities for biomedical exploitation [161], such as novel therapeutics for Alzheimers disease [162] or approaches for improving nerve repair [163].
